# Neurotoxicity of Nanomaterials: An Up-to-Date Overview

**DOI:** 10.3390/nano9010096

**Published:** 2019-01-13

**Authors:** Daniel Mihai Teleanu, Cristina Chircov, Alexandru Mihai Grumezescu, Raluca Ioana Teleanu

**Affiliations:** 1 Emergency University Hospital, Bucharest, Romania, “Carol Davila” University of Medicine and Pharmacy, Bucharest 050474, Romania; telepapa@hotmail.com (D.M.T.); raluca.teleanu@umfcd.ro (R.I.T.); 2 Faculty of Engineering in Foreign Languages, Politehnica University of Bucharest, București 060042, Romania; cristina.chircov@yahoo.com; 3 Department of Science and Engineering of Oxide Materials and Nanomaterials, Faculty of Applied Chemistry and Materials Science, Politehnica University of Bucharest, Bucharest 060041, Romania; 4 ICUB—Research Institute of University of Bucharest, University of Bucharest, 36-46 M. Kogalniceanu Blvd., Bucharest 050107, Romania

**Keywords:** neurotoxicity, nanomaterials, drug delivery, bioimaging, gene therapy, cancer therapy

## Abstract

The field of nanotechnology, through which nanomaterials are designed, characterized, produced, and applied, is rapidly emerging in various fields, including energy, electronics, food and agriculture, environmental science, cosmetics, and medicine. The most common biomedical applications of nanomaterials involve drug delivery, bioimaging, and gene and cancer therapy. Since they possess unique properties which are different than bulk materials, toxic effects and long-term impacts on organisms are not completely known. Therefore, the purpose of this review is to emphasize the main neurotoxic effects induced by nanoparticles, liposomes, dendrimers, carbon nanotubes, and quantum dots, as well as the key neurotoxicology assays to evaluate them.

## 1. Introduction

In general terms, nanotechnology is a multidisciplinary field which aims to control matter at the atomic and molecular levels [1,2]. Furthermore, it is defined as “the design, characterization, production, and application of materials, devices, and systems by controlling shape and size at the nanoscale”, comprising both the process, also known as nanofacture or ultraprecision engineering, and the class of materials [3,4]. Owing to its tremendous capacity to improve the performance of many different areas of science, it has been widely applied in various fields, including energy, electronics, food and agriculture, environmental science, cosmetics, and medicine [1,5,6,7,8,9].

Recently, novel terms have been employed due to emerging nanotechnology applications. Therefore, terms such as nanobiotechnology and bionanotechnology, which represent the application of nanotechnology to biology and the application of biology to nanotechnology, respectively, have arisen. Both terms encompass nanomedicine, defined as the application of nanotechnology to human health [3].

Moreover, most nanomaterials are defined as insoluble or biopersistent [10,11], naturally occurring or intentionally manufactured materials with at least one dimension or an internal structure within the nanoscale regime, specifically within 100 nm [12,13,14]. Nevertheless, nanomaterials are characterized by unique, fascinating, and useful properties, since their synthesis does not simply imply the change of bulk materials’ dimensions [13,15]. Unlike microscale materials, nanomaterials exhibit enhanced properties, owing to the large surface-to-volume ratio and the quantum confinement effect [12,16,17].

**Figure 1:**
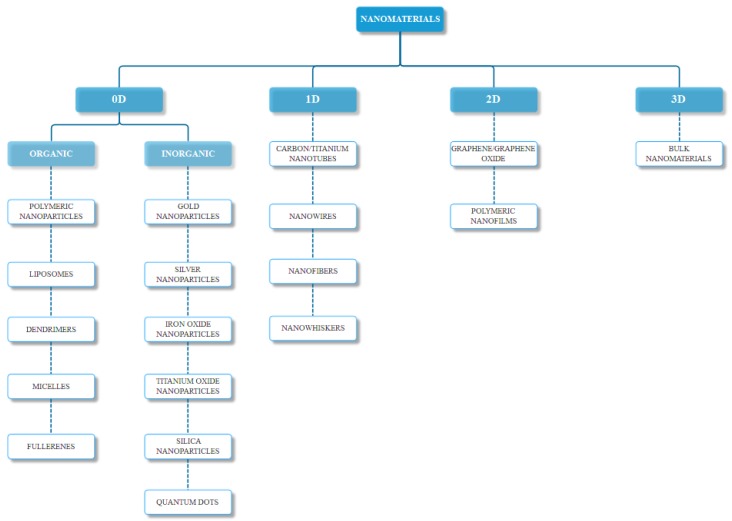
Nanomaterials classification based on their dimensionality (0D—zero-dimensional, 1D—one-dimensional, 2D—two-dimensional, 3D—three-dimensional).

As previously mentioned, nanomaterials can occur naturally, or they can be chemically, physically, mechanically, or biologically synthesized. The most common classification is based on their structure, namely zero-dimensional, one-dimensional, two-dimensional, or three-dimensional (Figure 1), with some materials falling on the borders of these categories. Furthermore, there are several other parameters for the classification of nanomaterials, including their chemical composition (organic and inorganic), their formation (biogenic, geogenic, anthropogenic, and atmospheric), their size and shape, and their application in industry or research [18].

To date, because nanomaterials can act as Trojan horses, they have been used as carriers for the delivery of drugs and imaging probes to specific cells and tissues [19]. Tremendous efforts have been made in developing nanomedicines for the diagnosis and therapy for the effective treatment of various brain diseases, including brain cancer, Alzheimer’s disease, Parkinson’s disease, amyotrophic lateral sclerosis, multiple sclerosis, and ischemic stroke [20,21,22]. A variety of nanomaterials have been applied for brain diseases therapy, including polymeric nanoparticles, liposomes, dendrimers, metallic nanoparticles, carbon nanotubes, and quantum dots [23,24].

However, in addition to the intentional application of nanomaterials for brain disorders therapy, they can unintentionally enter the brain through blood circulation from peripheral disorders therapy or after inhalation from the air. Therefore, in spite of the impressive progress in the field, the application of nanomaterials has brought about a new cause for concern, which is the neurotoxicity of nanomaterials [25]. As they can enter the brain by penetrating the blood–brain barrier through a series of transporters or receptors expressed on the endothelial cells of brain capillaries, through adsorptive-mediated transcytosis, or by bypassing the blood–brain barrier through the intranasal route, neurotoxicity must be taken into consideration in the manufacturing process [26]. The common neurotoxicity mechanisms involve oxidative stress, induced cell apoptosis and autophagy, and immune response and inflammation, activating specific signaling pathways which will subsequently affect the blood–brain barrier functions [27]. Moreover, a neurotoxic effect can directly alter the neuronal structure or activity, or it can result in a cascade of effects due to glial activation and glial–neuronal interactions. It can manifest immediately or after longer periods of time. The consequences of neurotoxicity can be reversible or permanent, affecting parts of the central nervous system or the whole system [28].

Conventional methods and techniques used to analyze the toxicity of nanomaterials are not sufficiently accurate and reliable, and it is not safe to draw conclusions regarding the potential dangers and mechanisms of the toxicity of nanomaterials [29]. Hence, there is a need to improve the novel discipline that combines nanotoxicology and neuroscience by dedicating original and specific experimental models and tools, as it is essential for studying the interactions of nanomaterials with the brain [30]. Elucidating and understanding neurotoxicity processes represents a way to design safer nanocarrier systems and reduce their side effects [31].

## 2. Toxicity Assessment of Nanomaterials

The interface between toxicology and nanomaterials has led to the emergence of a sub-discipline, namely nanotoxicology, to better reflect the unique physicochemical properties of nanomaterials [32]. Therefore, from the biomedical perspective, nanotoxicology refers to the biological effects caused by the specific characteristics of nanomaterials. Additionally, toxicity assessments of nanomaterials should follow a standardized set of rules [33] to avoid confusion and misconduct in designing nanomaterials for biomedical applications. Generally, the direction for toxicity assessment subsequently involves the characterization of nanomaterials, in vitro and in vivo studies, and final clinical trials.

### 2.1. Nanomaterial Characterization

To improve the quality and relevance of toxicological studies, standardized guidelines should be implemented on the physicochemical characterization of nanomaterials. Furthermore, the information acquired in this step should be relevant to the end-points of the study [34]. There are several essential physicochemical characteristics to be studied, including mean size and size distribution, surface area, chemical composition, surface charge and reactivity, shape, solubility, aggregation tendency in relevant media, crystallinity, porosity, and sample purity [34,35,36].

However, there is an important discrepancy between the physicochemical characteristics of nanomaterials in cell-free media and the conditions inside the organism [20]. Moreover, nanomaterials interact with body components, especially through protein adsorption, which can subsequently result in changes in nanomaterial surface characteristics. Consequently, combination with biological macromolecules can promote cellular uptake that will reduce body clearance and lead to chronic and degenerative changes [37]. Therefore, nanomaterials should be characterized both as manufactured, in the pristine state, and as administered, in the applied form [38].

Nanomaterial characterization is generally achieved through optical spectroscopy, electron microscopy, X-ray diffraction, light scattering, magnetic resonance, mass spectrometry, chromatography, zeta potential measurement, thermal techniques, and circular dichroism [39]. These techniques can be specific for certain properties, or they can be combined. There are several factors to consider when applying a certain technique, such as availability, selectivity, precision, non-destructive nature, cost, simplicity, and affinity to specific materials [40].

### 2.2. In Vitro Studies

In vitro toxicity assessments are crucial for investigating the mechanistic effects of nanomaterials on biological entities [41]. There are several advantages associated with in vitro tests, such as low costs, short time for acquiring results, and minimal ethical concerns [42]. Conventional in vitro models consist in monolayer cell cultures, cocultures, multilayer cocultures, xenograft models, and tissue slices, which are mostly employed for cytotoxicity analysis of various chemicals [43] and the impact of nanomaterials on cellular physiology [41]. To assess the neurotoxicity of nanomaterials, brain-specific cell types should be used, specifically blood–brain barrier and blood–liquid barrier cells, glial cells or neuroglia, and neurons with and without myelin sheaths [29], a membranous wrapping responsible for increasing the speed of signal conduction. The larger the neuron is, the thicker the myelin sheath is. Therefore, small neurons are unmyelinated, and the velocity for their signal conduction is very low [44]. Moreover, the use of unmyelinated neurons for in vitro neurotoxicity assessments is important because they are found in large numbers in pathologies associated with demyelination mechanisms, such as multiple sclerosis [45].

In vitro toxicity assessments can be categorized into proliferation, apoptosis, necrosis, oxidative stress, and DNA damage assays [42], and the common methods involve molecular, fluorescence, chemiluminescence, and analytical strategies [46]. Cell proliferation, defined as the increase in cell number secondary to cell growth and division, can be assessed through the main aspects of cell division, namely the nucleoside-analog incorporation during DNA synthesis, the cell cycle-associated proteins, and the cytoplasmic proliferation dyes [47]. As disorders of neuronal proliferation have a major impact on the function of the brain [48], leading to the development of various diseases and carcinogenesis, assessing the cellular proliferation in the presence of nanomaterials is of key importance [49]. Additionally, assessing cell death, which typically occurs through apoptosis or necrosis, is an essential step in determining the neurotoxic effects of nanomaterials. Apoptosis, the programmed cell death, is a physiological process characterized by changes in the nuclear morphology owing to chromatin fragmentation and condensation, the occurrence of apoptotic bodies, and cell shrinkage [50], through which cells are removed from tissues in a controlled manner. The main mechanisms for apoptosis involve caspase activation, cellular protein substrates cleavage, and DNA fragmentation [51]. By contrast, necrosis represents accidental cell death, occurring without any underlying signals as a consequence of non-specific cell injuries caused by trauma, hypoxia, or pathogens [52]. The characteristics of necrotic cells, including nuclear swelling, chromatin flocculation, loss of organelle function, membrane break, and cytolysis, can be studied through microscopic techniques [50]. Another necessary assay involves the detection of oxidative stress as a response to the exposure of cells to nanomaterials. The in vitro assays involve the real-time or static detection of the mechanisms involving oxidative stress, namely the generation of free radicals, metabolites, and degradation products, or the perturbations of redox states [53]. Considering the high consumption of oxygen and polyunsaturated fatty acids, the abundance of redox-active transition metal ions, and the relatively low levels of reduced glutathione, which acts as an antioxidant in the elimination of free radicals, the brain is the organ most susceptible to oxidative stress [54]. As DNA is highly sensitive to oxidative stress and therapeutic drugs, the use of nanomaterials might lead to DNA lesions, which could result in genomic instability and disease development. Hence, assessing DNA damage is crucial for designing nanocarriers that pose no danger to the organism [46].

However, since cell cultures do not mimic the native tissue microenvironment and nanomaterials can interfere with the assay components, conventional cell-based assays provide unreliable information [55]. Therefore, novel strategies for designing in vitro models are necessary. Recently, great progress has been made in the field of organ culturing through three-dimensional (3D) bioprinting, organoids, and organs-on-a-chip [56].

### 2.3. In Vivo Studies

In vivo experiments are mandatory for investigating the neurotoxicity of nanomaterials because they allow for whole organ systems studies, which cannot be modeled in vitro [57]. Additionally, in vivo models are used to determine the unintentional brain uptake and the potential toxic effects of nanomaterials designed for the therapy of peripheric diseases.

Several in vivo models and techniques have been employed to assess the brain uptake of nanomaterials. Since the blood–brain barrier is relatively similar in all animals, with species-specific differences [58], the most common in vivo models include drosophila, zebrafish, rodent, canine, and non-human primate models. Moreover, the techniques for studying the brain uptake of drugs are categorized into invasive techniques, specifically in situ brain perfusion, intravenous injection, microdialysis, brain uptake index, cerebrospinal fluid sampling, blood/plasma ratio determination, and quantitative autoradiography, as well as non-invasive techniques, mostly involving imaging techniques [59].

Assessing the neurotoxic potential of nanomaterials primarily involves the exploration of certain clinical signs (including changes in behavior regarding movement, learning, memory, motor coordination or reflexes, tremor, or paralysis), neurochemical signs (such as neuropathies and the synthesis, release, and uptake of neurotransmitters), neurophysiological signs, and neuroanatomical effects [60].

## 3. Neurotoxicity of Nanomaterials

**Figure 2:**
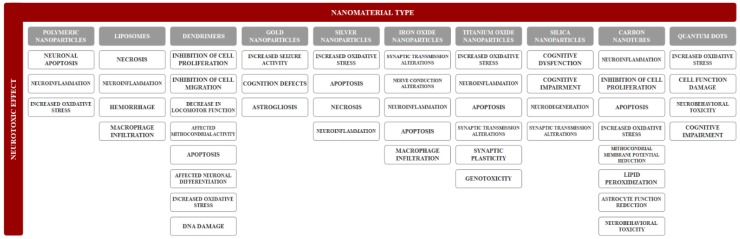
A summary of nanomaterial-induced neurotoxic effects.

Neurotoxicity refers to any reversible or irreversible adverse effect on the structure, function, or chemistry of the nervous system, during development or at maturity, produced by physical or chemical causes. Moreover, an adverse effect represents any change caused by treatment administration that affects the normal function. Common neurological adverse effects (Figure 2) regarding morphological changes involve neuronopathy, axonopathy, myelinopathy, and gliopathy [61]. The main mechanisms for neurotoxicity involve the excessive production of reactive oxygen species leading to oxidative stress, the release of cytokines causing neuroinflammation, and dysregulations of apoptosis leading to neuronal death [62]. However, there is a considerable lack of information regarding the neurotoxicity of nanomaterials, which complicates risk assessments following exposure. Therefore, the development of rapid, accurate, and effective strategies for determining the neurotoxic effects induced by nanomaterials is crucial [63].

### 3.1. Organic Nanomaterials

#### 3.1.1. Polymeric Nanoparticles

Polymeric nanoparticles, one the most studied organic nanomaterials in nanomedicine [64], have recently attracted great interest owing to their outstanding properties and behaviors in diagnosis and drug delivery applications. They possess a series of advantages, including controlled release, specific targeting, and the ability to protect drug molecules and to simultaneously diagnose and treat various diseases [65]. However, the disadvantages of polymeric nanoparticles involve aggregation and potential toxicity associated with the degradation processes and their residual materials [66].

Although there are a great number of studies regarding the use of polymeric nanoparticles for the diagnosis and/or therapy of brain diseases, neurotoxicological studies are limited. One study focused on the in vivo neurotoxicity evaluation of Polysorbate 80-modified chitosan nanoparticles after intravenous injection in rats. Results showed a dose-dependent accumulation of the nanoparticles in the frontal cortex and cerebellum, with neuronal apoptosis, slight inflammatory response, increased oxidative stress, and body weight loss [67]. Furthermore, the neurotoxic effects after exposure to polybutylcyanoacrylate nanoparticles as drug delivery systems across the blood–brain barrier have been evaluated. Although the in vitro results showed an increase in cell death associated with a high dosage of nanoparticles, the injection of the same dosage in rats did not produce any neuronal death. Therefore, the potential of this system for brain diseases therapy was proven [68].

#### 3.1.2. Liposomes

Liposomes are artificial, nanosized to microsized vesicles [69], consisting of an aqueous solution core surrounded by one or more amphiphilic lipid bilayers [70,71,72]. Consequently, liposomes can encapsulate both hydrophilic therapeutics in the aqueous core and hydrophobic molecules in the phospholipid bilayers [70,71]. Therefore, they have been extensively used in formulations as nanocarriers for the effective delivery of drugs, vaccines, proteins, enzymes, nucleic acids [70], and imaging agents [73]. Recent works have demonstrated their potential to deliver therapeutic and diagnostic agents to the brain, across the blood–brain barrier, through passive or active targeting [74].

One study reported the in vivo evaluation of the anti-cancer therapy efficiency of cisplatin-containing liposomal formulations and the associated neurotoxic effects of drug-free liposomes. Results showed an increased in vitro cytotoxicity against glioma cells and high tumor retention in glioma-bearing rats compared to the free cisplatin. However, the administration of the drug-free liposomes induced minimal to severe neuropathologic changes in the control rats, specifically neuroinflammation and necrosis. Moreover, administration of a commercially available drug-free liposomal formulation or a formulation containing lower doses of cisplatin induced mild to severe hemorrhage, necrosis, edema, and macrophages infiltrates. Therefore, it can be assumed that the neurotoxicity was due to the intrinsic toxicity of the liposomes combined with the neurotoxic effects of cisplatin [75].

#### 3.1.3. Dendrimers

Dendrimers represent a class of artificial, highly branched globular macromolecules with a tree-like topological structure, with sizes within the nanoscale [76,77]. They consist of a core, branched repeat units emerging from the core, and functional end groups on the outside layer of the repeat units [76]. The most common molecules used as basic units are polyamidoamine, polypropylenimine, and poly(aryl ether) [77]. These matchless polymer-based nanostructures [78] have the capacity to entrap both hydrophilic and hydrophobic molecules; thus, they are used as nanocarriers for therapeutic and imaging agents [77,79]. Moreover, they have been extensively used for brain diseases therapy as they can overcome the blood–brain barrier [80].

Depending on their physicochemical characteristics, such as surface chemistry, surface charge, and size, dendrimers have been shown to induce several neurotoxicological responses inside the organism [81].

The effects on a 3D neurosphere system using human neural progenitor cells after exposure to polyamidoamine dendrimers were evaluated. Results showed that higher concentrations induced significant inhibition of cell proliferation and migration [82]. Furthermore, dendrimers of different generations were used in zebrafish embryos and larvae to assess the associated toxic effects. An innate immune response was observed in the embryos, suggesting a time- and concentration-dependent toxicity. Moreover, the neurotoxic effects could be associated with the decrease of the locomotor function of the larvae when administering the lower generation dendrimers [83]. Similarly, dendrimers of different generations with various surface groups were used to assess the neurotoxic effects on human neural progenitor cells. Results showed that cationic dendrimers of higher generations affected mitochondrial activity, apoptosis, neuronal differentiation, and gene expression related to oxidative stress and DNA damage. Additionally, results demonstrated that the number of particles and the surface group density are more important characteristics to influence the cytotoxicity of cationic dendrimers [84]. Surface functionalization with polyethylene glycol or folate to reduce the neurotoxicity of dendrimers has been studied. Results confirmed the potential of this strategy to prevent synaptic dysregulation and cell viability decrease associated with the neurotoxicity of the unmodified dendrimers [85].

### 3.2. Inorganic Nanomaterials

#### 3.2.1. Inorganic Nanoparticles

Recent studies have proved the capacity of inorganic nanoparticles, such as gold, silver, iron oxide, titanium oxide, and silica nanoparticles, to translocate into the brain after entering the body. Furthermore, due to their limited excretion, they gradually accumulate in the brain, causing damage to neuronal cells and function impairments [86].

After entering the brain by crossing the blood–brain barrier or along the olfactory nerves, gold nanoparticles accumulate in the brain where they induce neurotoxic effects. The main neuropathological symptoms caused by gold nanoparticles involve increased seizure activity, cognition defects, and astrogliosis [87], which represents a change in the morphology of astrocytes, forming reactive astrocytes. Astrogliosis is most common in brain diseases associated with hypoxia, ischemia, and seizures [88].

Assessing the neurotoxic effects of silver nanoparticles is fundamental, considering the extensive application in a variety of fields, including food, pharmacological, and environmental industries, medicine, cosmetics, and textile coatings [89]. Recent studies have indicated the potential role of nanoparticle size, shape, surface coatings, release rates of silver ions, and interactions with specific cells and proteins on their neurotoxicity [90]. Furthermore, the mechanisms for neurotoxicity involve the cellular internalization of silver nanoparticles, leading to intracellular reactive oxygen species generation and promoting cell death. Nonetheless, lower levels of silver nanoparticles induced an increase in caspase activity and cytokines secretion from astrocytes, leading to apoptosis and neuroinflammation, respectively. Additionally, the release of silver ions produced damage to cell membrane integrity and caused cell necrosis [91].

Owing to their unique magnetic properties and biodegradability, iron oxide nanoparticles have been increasingly employed in the field of medicine, for imaging, drug delivery, cancer therapy, and cell separation purposes [92]. Following the rapid developments in physics, chemistry, biology, and nanotechnology in the past years, physicochemical tailoring and surface modification have enhanced the efficiency of iron oxide nanoparticles [93], but have also raised concerns regarding their neurotoxicity. The interactions of iron oxide nanoparticles with cellular components are determined by the oxidation state of iron and the concentration, size, coating, and functional groups of the nanoparticles [94]. Studies reported the risks associated with the exposure to iron oxide nanoparticles, namely alterations in synaptic transmissions and nerve conduction, leading to neuroinflammation, apoptosis, and immune cell infiltration [95].

Titanium oxide nanoparticles are the most common nanomaterials used in biomedicine, cosmetics, and as food supplements in chewing gums, candies, and toothpastes. Therefore, their neurotoxicity must be assessed since the routes for entering the brain involve the blood–brain barrier pathways and occur along the olfactory and taste nerves [96]. The main mechanisms of neurotoxicity induced by exposure to titanium oxide nanoparticles involve oxidative stress, neuroinflammation, apoptosis, genotoxicity, dysregulated neurotransmitters, synaptic plasticity, and dysrupted signaling pathways [97].

Recent studies have focused on the role of silica nanoparticles in neurodegeneration. The intranasal route leads to the accumulation of nanoparticles in the brain and subsequently to cognitive dysfunction and impairment, synaptic changes, and pathologies similar to neurodegeneration [98]. Furthermore, other studies reported the neurotoxic effects caused by administering low concentrations of silica nanoparticles, specifically the increase in oxidative stress and alterations of microglial functions, leading to highly negative impacts on the striatum and dopaminergic neurons [99].

#### 3.2.2. Carbon Nanotubes

Owing to their unique structure, high surface area, and excellent electrical, mechanical, optical, and thermal properties, carbon nanotubes have attracted great interest in various fields [100,101]. Among the carbon-based nanomaterials, carbon nanotubes are the most commonly used nanomaterials in the field of nanomedicine for drug, hormone, and enzyme delivery, gene therapy, and tissue engineering and as biosensors, nanoprobes, and nanorobots [101,102]. Nevertheless, their application in biomedicine has incited a cause for concern regarding their toxicity to biological tissues [102].

Carbon nanotubes enter the brain through olfactory or systemic administration. Studies have shown that the inhalation of carbon nanotubes leads to their accumulation in the olfactory bulb, resulting in inflammatory responses by the activation of microglial cells. The factors causing the neurotoxic effects of carbon nanotubes are their diameters or lengths, structures, concentration, and impurities [103]. Studies have reported that multi-walled carbon nanotubes induce higher neurotoxic effects than single-walled carbon nanotubes [104]. Furthermore, longer carbon nanotubes in higher concentrations are more neurotoxic when compared to their counterparts [103].

Common neurotoxic effects induced by carbon nanotubes exposure involve inhibition of cell proliferation, apoptosis, mitochondrial membrane potential reduction, promoting reactive oxygen species formation, lipid peroxidization, and astrocyte function reduction [103]. Moreover, carbon nanotubes might cause neurobehavioral toxicity, including anxiety and depression [104].

#### 3.2.3. Quantum Dots

Quantum dots have gained considerable scientific interest in the past years due to their unique optical and electrical properties [105]. These zero-dimensional nanomaterials have generated great potential for novel applications in biology and medicine, including drug delivery, cell labeling and tracking, targeted cancer therapy, and bioimaging [106]. Assessing their toxicity, especially their neurotoxicity, is fundamental for their emerging application in the biomedical field.

The neurotoxic effects of quantum dots are dependent on their size, surface charge, concentration, surface coating, the nature and the solubility of the constituent materials, and purity [107,108]. Furthermore, several neurotoxic effects are similar to the general toxicity, including increased oxidative stress and cell function damages, whereas behavioral changes and learning and memory impairments are specific to neurotoxicology [108]. Moreover, when assessing the neurotoxicity of quantum dots, dosage, administration route, concentration, duration of exposure, distribution, metabolism, and excretion represent fundamental aspects of the study [108]. There are several strategies to reduce the neurotoxicity and to regulate the behavior of quantum dots, such as surface modification through zinc sulfide coating [107].

## 4. Conclusions and Perspectives

Nanomaterials are increasingly applied in the field of biomedicine for drug delivery, bioimaging, and gene and cancer therapy. However, since they can unintentionally enter the body and subsequently the brain, the potential neurotoxic effects of nanomaterials must be assessed. Although there are several available toxicity assessments, including nanomaterial characterization and in vitro and in vivo studies, there is a lack of standardized and reliable neurotoxicological studies. Since any type of nanomaterial has shown at least a minimum level of neurotoxicity, a thorough evaluation is crucial for designing safer nanocarrier systems and reducing their side effects. Furthermore, strategies to reduce the neurotoxicity, including the removal of toxic materials from the composition, reducing the period of exposure, controlling the size and shape, and coating the nanomaterials to modify the surface properties, must be employed.
